# Supplementary material for: Low-Dose Delta-9-Tetrahydrocannabinol as Beneficial Treatment for Aged APP/PS1 Mice
Source: Int J Mol Sci. 2022 Mar 2;23(5):2757. doi: 10.3390/ijms23052757 (PMC8910894; doi:10.3390/ijms23052757)
Supplement: Supplementary file 1 [file ijms-23-02757-s001.zip › ijms-1582420-supplementary.pdf]

Supplemental Materials

Low-Dose Delta-9-Tetrahydrocannabinol as Beneficial Treatment for Aged APP/PS1 Mice

Yanhong Wang[a], Yuzhu Hong[a], Jiyu Yan[a], Breanna Brown[a], Xiaoyang Lin[a], Xiaolin Zhang[b], Ning Shen[a,d], Minghua Li[a], Jianfeng Cai[d], Marcia Gordon[c], David Morgan[c], Qingyu Zhou[a,*,] and Chuanhai Cao[a,b,*]

Table S1. Information of individual mice used in the present study

Group	Mouse ID	Sex	Average Latency in the Last Trial (seconds)	Average Number of Errors in the Last Trial	Memo
NTG Control	MO01 222A-3	Female	6.28	0.667	
	MO04 223A-2	Female	14.48	0.667	
	MO10 269A-2	Female	23.72	1	
	MO05 223A-8	Male	34.71	5	
	MO06 285A-2	Male	15.15	1	
	MO11 217A-4	Male	17.25	1.67	
	MO12 288A-10	Male	21.26	1.67	
	MO02 205B-1	Male	4.12	1	Found dead
	MO07 296A-2	Male	20.51	1.67	Euthanized due to skin ulcer
	MO08 288A-3	Female	10.63	1.33	Found dead
	MO18 213A-2	Female	22.14	2	Found dead
TG Control	MO15 161A-7	Female	59.22	6	
	MO39 217A-6	Female	42.17	3.33	
	MO41 296A-10	Female	56.33	8	
	MO20 285A-1	Male	28.23	2.67	
	MO23 203B-4	Male	33.13	2.33	
	MO32 185A-9	Male	40.69	5	
	MO40 296A-6	Male	30.07	3	
	MO17 205B-2	Male	21.61	3.33	Found dead
TG THC 0.02 mg/kg	MO14 161A-6	Female	26.54	2.67	
	MO22 203B-1	Female	34.48	3	
	MO26 233A-4	Female	35.05	4.33	
	MO34 206A-7	Female	29.66	3.67	
	M036 199B-1	Female	38.41	5	
	MO24 220A-3	Male	49.39	4.33	
	MO27 269A-1	Male	46.82	3.33	
	MO29 179B-2	Male	41.87	7	
	MO31 185A-5	Male	28.96	3	
	MO21 198B-5	Male	60	5.67	Found dead
TG THC 0.2 mg/kg	MO13 161A-4	Female	29.93	4	
	MO16 222A-1	Female	31.71	3.67	
	MO30 185A-3	Female	59.97	5.33	
	MO37 188B-2	Female	60	5.33	
	MO19 213A-10	Male	32.89	3	
	MO35 266A-2	Male	58.93	4.33	

	MO38 217A-2	Male	55.89	4.33	
	MO25 224A-1	Female	57.80	6	Euthanized due to intestinal tumor
	MO28 288A-6	Male	30.87	3	Found dead
	MO33 206A-2	Male	36.17	3.67	Found dead

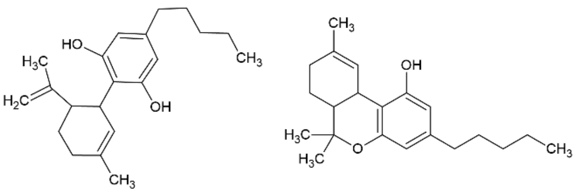

Cannabidiol (CBD) Tetrahydrocannabinol (THC)

$C_{21}H_{30}O_2$ $C_{21}H_{30}O_2$

Containing hydroxyl group Containing cyclic ring

Figure S1. Structures of cannabidiol (CBD and Δ-9-tetrahydrocannbinol (THC)

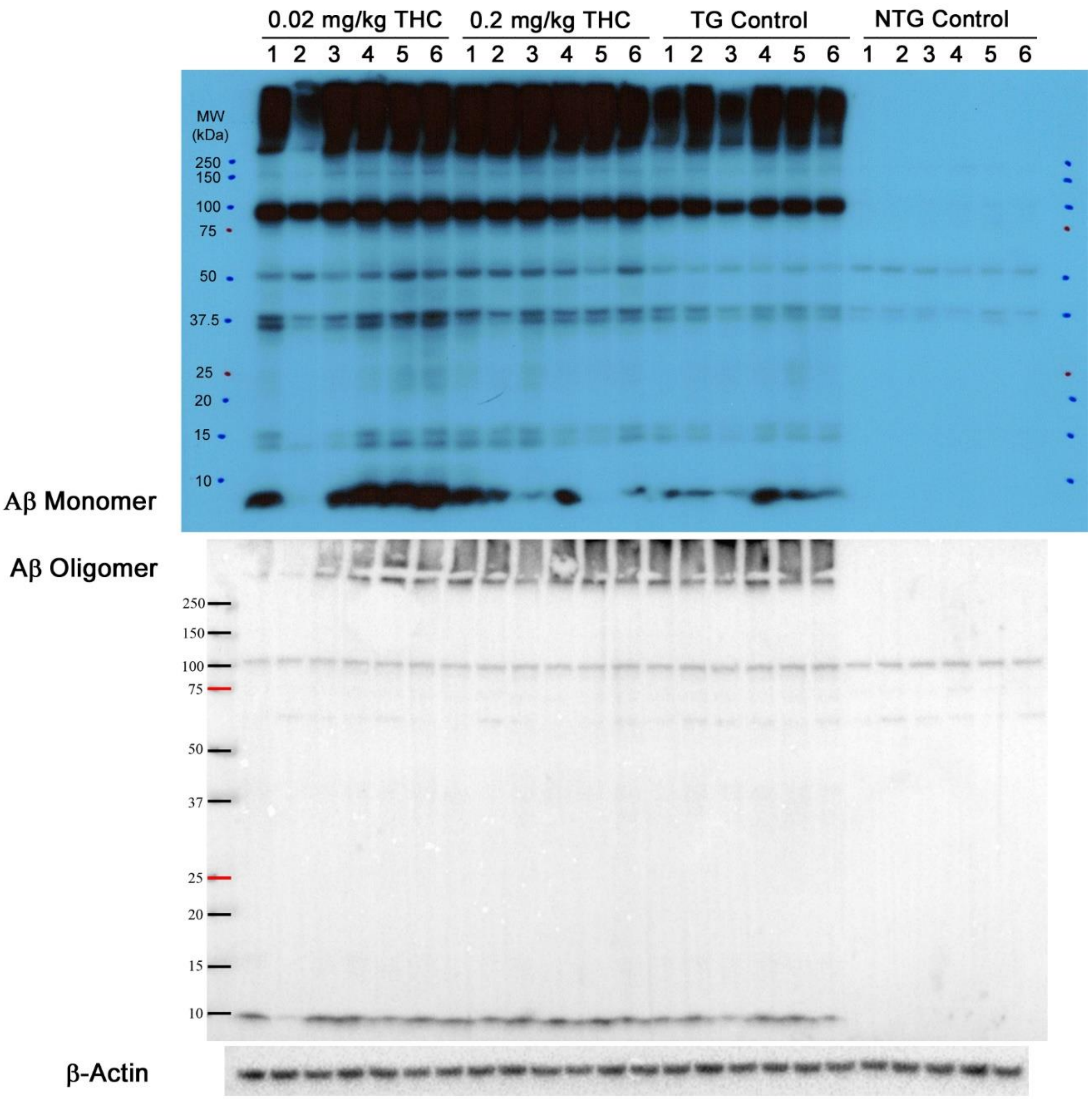

Figure S2. Western blot images of the expression of Aβ monomers and Aβ oligomers in individual brain homogenate samples collected from control non-transgenic (NTG) (N = 6), control transgenic APP/PS1 (TG), 0.02 mg/kg (N = 6) and 0.2 mg/kg (N = 6) THC treatment groups. β-Actin served as the loading control.
